# A national cross-sectional study on latent profile analysis of occupational fatigue among Chinese nurses in the early post-COVID-19 era

**DOI:** 10.3389/fpubh.2024.1501417

**Published:** 2025-01-07

**Authors:** Qiuyang He, Jianhua Ren, Guoyu Wang, Yonghong Wang

**Affiliations:** ^1^Department of Obstetric Nursing, West China Second University Hospital, Sichuan University, Chengdu, China; ^2^Key Laboratory of Birth Defects and Related Diseases of Women and Children (Sichuan University), Ministry of Education, Chengdu, China

**Keywords:** occupational fatigue, latent profile analysis, post-COVID-19 era, nurse, China

## Abstract

**Background:**

Occupational fatigue is a widespread condition within the nursing workforce, adversely affecting both nurses’ health and patient safety. The protracted duration of the COVID-19 pandemic, approaching 3 years, has exacerbated the challenges faced by nurses globally. The escalation in patient numbers and the high incidence of infections among healthcare workers have intensified occupational fatigue. This study seeks to explore the enduring impact of the pandemic on occupational fatigue among Chinese nurses through a latent profile analysis, and to identify the associated risk factors.

**Methods:**

A comprehensive survey was conducted involving 2,140 nurses from 186 hospitals across China during the initial phase of the post-COVID-19 era. The primary instruments utilized for data collection were the Occupational Fatigue Exhaustion Recovery Scale and the Effort-Reward Imbalance Questionnaire.

**Results:**

Three distinct profiles of occupational fatigue were identified: a low fatigue/high recovery group (18.6%), a moderate fatigue/moderate recovery group (48.8%), and a high fatigue/low recovery group (32.6%). The vast majority of nurses reported experiencing moderate to high levels of occupational fatigue during the early stage of the post-coronavirus era. Significant predictors for membership in these fatigue profiles included marital status, possession of a master’s degree or higher, working over five night shifts per month, experiencing COVID-19 symptoms, and exhibiting higher scores in extrinsic effort and overcommitment.

**Conclusion:**

Chinese nurses exhibit a relatively high level of occupational fatigue in the early post-COVID-19 era, likely influenced by various socio-demographic and work-related factors. It is imperative to develop targeted interventions aimed at alleviating fatigue among specific groups of nurses to effectively address the challenges posed by occupational fatigue in the face of future public health disasters.

## 1 Introduction

Occupational fatigue stands out as a predominant public health concern affecting the working population. It is characterized as a self-recognized state emerging in individuals confronting with excessive demands and stressors from their working environment, tasks, and schedules, coupled with inadequate recovery. This condition hampers workers’ labor ability as a result of the physical and/or mental exhaustion (1). Nurses, as the largest occupational group within health sectors, bear a crucial responsibility for ensuring patient safety. Their role involves detecting errors and near misses, recognizing and communicating changes in patients’ condition, and delivering high-quality health care (2). Given the inherent challenges of their profession, nurses regularly contend with extended working hours, unpredictable surges in workloads, and emotional and ethical complexities. Consequently, nurses are considered a high-risk group for occupational fatigue. Previous research indicates that nurses may experience heightened fatigue levels compared to other healthcare professionals due to their more frequent and closer interaction with patients (3–5). Fatigued nurses are more prone to experience adverse effects on both their own health and patients’ safety. These effects encompass workplace injuries, delayed reaction time, decision regret, increased turnover intention, and a higher incidence of medical errors (6). Recognizing the significance of this issue, various professional organizations, such as the American Nurses Association (ANA), the Joint Commission Institution (JCI), and the Institute of Medicine (IOM) have prioritized addressing and alleviating professional fatigue among nurses as a crucial step in enhancing healthcare quality (6–8).

With the advent of the coronavirus disease 2019 (COVID-19), the workload for nursing staff has witnessed a staggering increase. This surge in responsibilities encompasses new demands for professional protection, frequent training on rapidly updated protocols, an influx of emergency and critically-ill patients, and the navigation of unfamiliar settings, specifically tailored to address the challenges posed by COVID-19. Substantial research indicates that a considerable proportion of nurses experienced heightened levels of fatigue during the peak of the pandemic (9–11). In December 2022, China officially terminated the policy of domestic isolation and lock-downs, subsequently opening its borders from January 8, 2023, marking the commencement of the post-COVID-19 era (12). The unique transition period in post-COVID-19 has posed distinct challenges to the healthcare system, including the sudden surge in patient numbers and a significant increase in infections among healthcare workers (13). Undoubtedly, Chinese nurses are grappling with tremendous stress during this period, further exacerbating occupational fatigue. Recognizing the importance of post-disaster reviews, the Sendai Framework for Disaster Risk Reduction emphasizes their role in providing valuable lessons for the public health system and enhancing disaster preparedness (14). In light of these considerations, there is a pressing need to explore and understand the impact of the post-COVID-19 era on occupational fatigue of Chinese nurses in this critical period.

Despite the repeated emphasis on the importance of understanding the long-term influences of the pandemic on occupational fatigue, studies in this area remain limited (15–17). Moreover, existing research predominantly adopts a variable-centered approach, examining various domains of occupational fatigue separately (15, 16). Several limitations are intrinsic to this approach, as it treats participants as a homogeneous group and overlooks potential variations in relationships among variables within subgroups of participants. To address these limitations, our present study employs a person-centered method known as Latent profile analysis (LPA). Unlike the variable-centered approach, LPA utilizes continuous explicit variables to cluster data, enabling the exploration of heterogeneity within populations (18). This approach, centered on individuals, places emphasis on the identification of latent subgroups of participants through the utilization of observed indicators, providing a greater degree of specificity (19). In our study, we applied LPA to cross-sectional survey data to investigate the status of occupational fatigue among Chinese nurses in the early stage of the post-COVID-19 era and identify associated risk factors. The results of this analysis can serve as valuable insights for policy makers and nursing administrators, aiding in the development of optimal fatigue management strategies tailored to the specific needs and challenges faced by Chinese nurses at this crucial period.

## 2 Methods

### 2.1 Setting and participants

This nationwide survey targeted nurses employed in 186 hospitals across 28 provinces in China. Data collection occurred immediately following the termination of strict epidemic control and prevention policies spanning from January 17 to March 16 2023, representing the early stage of the post-COVID-19 era. A convenient sampling was applied for subjects recruiting, adhering to specific inclusion and exclusion criteria.

Inclusion criteria: (1) registered nurses delivering direct or indirect patient care in any nursing unit; (2) no known language, reading, or other cognitive problems. Exclusion criteria were: (1) nurses serving in management position (e.g., nurse manager, nurse administrator); (2) those currently under the supervision of an assigned preceptor; (3) nursing students and visiting scholars; (4) retired nurses; (5) nurses on vacation during the data collection period (e.g., maternity leave, medical leave, paid/unpaid leave); (6) participants engaged in other fatigue intervention studies.

The sample size determination involved the single population proportion formula:


$$N=Z\propto^{2}P\left(1-P\right)/d^{2}$$

Here, Zα is set at 1.96, representing the reliability coefficient at a 5% significance level. The acceptable margin of error (d) for the proportion was 3%, and the estimated prevalence (P) of nursing staff in China who experienced occupational fatigue during the COVID-19 pandemic was 72% (11). A minimum sample size of 860 was calculated to adequately represent the target population. To address the limitations inherent in convenience sampling and online surveys, particularly the clustering effect that may result in sample deviation and compromise the accuracy and reliability of study findings, a design effect (DE) factor was integrated into the equation to adjust for design discrepancies. Based on prior research, a DE of 2 was deemed the minimum acceptable level for convenience sampling studies (20, 21). Thus, an adjusted minimal sample size of 1720 (860 × 2) was confirmed.

### 2.2 Data collection

A self-reported online survey was used for data collection, providing subjects the flexibility of participating without constraints related to time and space. Formal communication was initiated with the Chinese Nursing Association to outline the research’s objectives and methodologies, seeking their collaboration in participant recruitment. It is noteworthy that this association holds the highest authority in representing the nursing profession comprehensively across China. Most of its members occupy roles such as directors of nursing departments or nurse managers, covering all regions of the country. The survey itself was administered using the online survey platform ‘Survey Star’. This platform was chosen for its accessibility and user-friendly interface, ensuring a smooth and efficient data collection process.

### 2.3 Measures

The survey tools were composed of two parts: the general information section and questionnaires addressing occupational fatigue and stress. The general information section gathered data on nurses’ personal health and demographic characteristics, encompassing gender, age, marital status, education, history of COVID-19 infection, current COVID-19 status, and disease progression. Work and COVID-19 patient care-related variables, including hospital sites and grades, years of work, professional title, practice unit, average worked hours per week, average number of night shifts per month, and direct provision of care to patients with COVID-19, were also assessed in this section.

The primary survey instruments employed were the Occupational Fatigue Exhaustion Recovery (OFER) Scale and the Effort-Reward Imbalance (ERI) Questionnaire. The OFER scale was mainly applied to measure work-related fatigue among nurses (22). It is a multidimensional instrument with three subscales, which are acute fatigue (OFER-AF), chronic fatigue (OFER-CF) and inter-shift recovery (OFER-IR). Each subscale consists of 5 items that are evaluated on a 7-point Likert scale, ranging from 0 (strongly disagree) to 6 (strongly agree), with items 9, 10, 11, 13 and 15 being reverse-coded. The total score for each subscale is determined by summing the scores of each item and dividing by 30, then multiplying by 100. This calculation yields a summative score varying from 0 to 100. Higher scores indicate higher levels of each dimension. The interpretation of different levels within each subscale is as follows: scores ranging from 0 to 25 are considered low, scores from 26 to 50 are classified as low-moderate, scores from 51 to 75 are categorized as moderate-high, and scores from 76 to 100 are regarded as high. Originally developed by Winwood et al. to assess work-related fatigue in nursing samples in Australia, the OFER scale demonstrated robust psychometric properties (23). The Chinese version, tested in healthcare workforce, including nurses, showed satisfactory psychometric properties with the content validity index as 0.92 and Cronbach’s alpha coefficient for the each subscale as 0.83, 0.85 and 0.86, respectively (24).

Occupational stress was assessed using the Effort-Reward Imbalance (ERI) Questionnaire, comprising three dimensions with a total of 23 items (25). These dimensions consist of extrinsic effort (6 items), reward (11 items), and overcommitment (6 items). Participants responded to the extrinsic effort and reward dimensions on a 5-point Likert scale (ranging from 1, indicating no stressful experience, and 5, indicating being very distressed by the typical experience). The overcommitment dimension utilized a 4-point Likert scale, ranging from 1 (strongly disagree) to 4 (strongly agree). Scores for each dimension varied from 6 to 30 (extrinsic effort), 11 to 55 (reward), and 6 to 24 (overcommitment), respectively. Higher scores were indicative of higher levels within each dimension. In accordance with the ERI model, an established algorithm calculated the extrinsic effort/reward ratio. The aforementioned ratio was obtained by dividing the effort score by the reward score, which was multiplied by a correction factor of 0.5454 to account for the number of items. This ratio serves as a measure of the potential incongruity between extrinsic effort and reward within the workplace. Values approaching zero indicate a favorable work environment characterized by relatively low effort and relatively high reward. Values exceeding 1.0 indicate a significant disparity between expended effort and received reward. Higher extrinsic effort/reward ratios and overcommitment scores denote elevated occupational stress (26). The ERI questionnaire, widely employed across various countries, has demonstrated excellent predictive validity and reliability in diverse working populations (27–29). It has also exhibited satisfactory psychometric properties in Chinese healthcare workers, including physicians and nurses, with Cronbach’s alpha coefficients for each subscale at 0.78, 0.81 and 0.74, respectively (30).

### 2.4 Statistical analysis

Latent profile analysis (LPA) was performed using Mplus version 8.3 to identify subgroups of hospital nurses with similar occupational fatigue profiles based on their responses to the OFER scale. A sequence of LPA models was estimated, with an increasing number of latent profiles. Iterative comparisons were conducted between each subsequent k-class model and the previous k-1 class models, using various statistical fit indices. These fit indices encompassed the Akaike Information Criterion (AIC), Bayesian Information Criterion (BIC), Sample Size-Adjusted BIC (aBIC), Lo–Mendell–Rubin Likelihood Ratio Test (LMRT), Bootstrap Likelihood Ratio Test (BLRT), and Entropy. Lower values of AIC, BIC, and aBIC indicate a finer balance between model fit and parsimony. Higher entropy values (close to 1) suggest better classification for the LPA model (31). If the LMRT test does not produce a statistically significant result (P>0.05), it is possible that a model with a smaller number of latent profiles may provide a better fit for the data (32). The determination of the optimal number of latent patterns was based on a comprehensive evaluation of fit statistics, theoretical assumptions, interpretability, and the distinct characteristics of the profiles. This comprehensive approach ensures the robustness and validity of the identified latent profiles in capturing the nuances of occupational fatigue among Chinese nurses in the early stage of the post-COVID-19 era.

All other statistical analyses were carried out with IBM SPSS 26.0. Continuous normal variables were reported as mean and standard deviation (SD), while categorical variables were presented as frequencies with percentage. To assess differences in the latent subgroups of occupational fatigue concerning nurses’ personal health and demographic characteristics, work and COVID-19 patient care variables, and occupational stress, one-way analysis of variance (ANOVA) and chi-square tests were employed. Multinomial logistic regression models were utilized to identify independent factors associated with occupational fatigue profile membership. A significance level of 0.05 (2-tailed) was adopted for all analyses.

## 3 Results

A total of 2,240 hospital nurses from 28 provinces in China were recruited, and 2,140 valid questionnaires were collected, yielding an effective response rate of 95.5%.

### 3.1 Socio-demographic and work-related characteristics of the participants

The participants had a mean age of 31.71 years, with an age range spanning from 20 to 59. On average, participants had 9.85 years of experience as registered nurses. The majority of nurses (82.3%) reported working over 40 h per week, and more than half (52.5%) indicated having more than 5-night shifts per month. Almost all respondents (94.7%) confirmed a history of COVID-19 infection. Further details regarding socio-demographic and work-related characteristics are presented in Table 1.

**Table 1:** Univariate analysis of the latent fatigue profiles against nurse characteristics (*N* = 2,140).

Characteristics	Groups	N (%)	Low fatigue/ High recovery (*n* = 398)	Moderate fatigue/ Moderate recovery (*n* = 1,044)	High fatigue/ Low recovery (*n* = 698)	χ^2^	*p-*value
Gender	Male	37 (1.7%)	5 (1.3%)	17 (1.6%)	15 (2.1%)	1.310	0.519
	Female	2,103 (98.3%)	393 (98.7%)	1,027 (98.4%)	683 (97.9%)		
Age in years	20 ~ 30	1,068 (49.9%)	191 (48.0%)	528 (50.6%)	349 (50.0%)	12.365	0.054
	31 ~ 40	873 (40.8%)	159 (39.9%)	414 (39.7%)	300 (43.0%)		
	41 ~ 50	159 (7.4%)	37 (9.3%)	78 (7.5%)	44 (6.3%)		
	>50	40 (1.9%)	11 (2.8%)	24 (2.3%)	5 (0.7%)		
Marital status	Single	595 (27.8%)	87 (21.9%)	307 (29.4%)	201 (28.8%)	12.702	0.013*
	Married	1,496 (69.9%)	297 (74.6%)	720 (69.0%)	479 (68.6%)		
	Divorced/widowed	49 (2.3%)	14 (3.5%)	17 (1.6%)	18 (2.6%)		
Education	Diploma	651 (30.4%)	132 (33.2%)	330 (31.6%)	189 (27.1%)	9.689	0.046*
	Bachelor’s degree	1,467 (68.6%)	260 (65.3%)	708 (67.8%)	499 (71.5%)		
	Master’s degree or above	22 (1.0%)	6 (1.5%)	6 (0.6%)	10 (1.4%)		
Hospital sites	Eastern China	134 (6.2%)	25 (6.3%)	61 (5.8%)	48 (6.9%)	35.138	<0.001*
	Central China	218 (10.2%)	67 (16.8%)	111 (10.6%)	40 (5.7%)		
	Western China	1788 (83.6%)	306 (76.9%)	872 (83.5%)	610 (87.4%)		
Hospital grades	Public tertiary hospital	1782 (83.3%)	349 (87.7%)	880 (84.3%)	553 (79.2%)	15.299	0.004*
	Public secondary hospital	275 (12.8%)	35 (8.8%)	127 (12.2%)	113 (16.2%)		
	Public primary hospital	83 (3.9%)	14 (3.5%)	37 (3.5%)	32 (4.6%)		
Years of work	<1	14 (0.7%)	2 (0.5%)	8 (0.8%)	4 (0.6%)	10.127	0.256
	1 ~ 5	628 (29.3%)	120 (30.2%)	310 (29.7%)	198 (28.4%)		
	6 ~ 10	699 (32.7%)	114 (28.6%)	344 (33.0%)	241 (34.5%)		
	11 ~ 20	636 (29.7%)	122 (30.7%)	300 (28.7%)	214 (30.7%)		
	>20	163 (7.6%)	40 (10.1%)	82 (7.9%)	41 (5.9%)		
Occupation	Nurse	1,564 (73.1%)	323 (81.2%)	761 (72.9%)	480 (68.8%)	19.812	<0.001*
	Midwife	576 (26.9%)	75 (18.8%)	283 (27.1%)	218 (31.2%)		
Professional title	Nurse	410 (19.2%)	78 (19.6%)	212 (20.3%)	120 (17.2%)	10.047	0.123
	Senior nurse	1,005 (47.0%)	192 (48.2%)	476 (45.6%)	337 (48.3%)		
	Supervisor nurse	665 (31.1%)	121 (30.4%)	317 (30.4%)	227 (32.5%)		
	Deputy chief nurse or above	60 (2.8%)	7 (1.8%)	39 (3.7%)	14 (2.0%)		
Practice unit	Outpatient department	263 (12.3%)	76 (19.1%)	140 (13.4%)	47 (6.7%)	70.512	<0.001*
	Emergency department	68 (3.2%)	18 (4.5%)	33 (3.2%)	17 (2.4%)		
	Operating room	102 (4.8%)	20 (5.0%)	48 (4.6%)	34 (4.9%)		
	Intensive Care Unit (ICU)	42 (2.0%)	9 (2.3%)	15 (1.4%)	18 (2.6%)		
	Internal medicine	132 (6.2%)	21 (5.3%)	43 (4.1%)	68 (9.7%)		
	Surgery department	103 (4.8%)	17 (4.3%)	47 (4.5%)	39 (5.6%)		
	Gynaecology department	169 (7.9%)	25 (6.3%)	84 (8.0%)	60 (8.6%)		
	Obstetrics department	984 (46.0%)	163 (41.0%)	493 (47.2%)	328 (47.0%)		
	Paediatrics department	228 (10.7%)	37 (9.3%)	119 (11.4%)	72 (10.3%)		
	Others (non-clinical departments)	49 (2.3%)	12 (3.0%)	22 (2.1%)	15 (2.1%)		
Average hours of weekly work	≤40	378 (17.7%)	113 (28.4%)	204 (19.5%)	61 (8.7%)	170.192	<0.001*
	41 ~ 45	906 (42.3%)	192 (48.2%)	470 (45.0%)	244 (35.0%)		
	46 ~ 50	497 (23.2%)	72 (18.1%)	221 (21.2%)	204 (29.2%)		
	>50	359 (16.8%)	21 (5.3%)	149 (14.3%)	189 (27.1%)		
Number of night shifts per month	0	498 (23.3%)	147 (36.9%)	249 (23.9%)	102 (14.6%)	87.830	<0.001*
	1 ~ 4	519 (24.3%)	103 (25.9%)	258 (24.7%)	158 (22.6%)		
	≥5	1,123 (52.5%)	148 (37.2%)	537 (51.4%)	438 (62.8%)		
Direct provision of care to patients with COVID-19	Yes	1904 (89.0%)	324 (81.4%)	927 (88.8%)	653 (93.6%)	38.177	<0.001*
	No	236 (11.0%)	74 (18.6%)	117 (11.2%)	45 (6.4%)		
History of COVID-19 infection	Yes	2027 (94.7%)	376 (94.5%)	998 (95.6%)	653 (93.6%)	3.543	0.170
	No	113 (5.3%)	22 (5.5%)	46 (4.4%)	45 (6.4%)		
Currently suffering from COVID-19 related symptoms	Yes	898 (44.3%)	86 (21.6%)	415 (39.8%)	397 (56.9%)	147.466	<0.001*
	No	1,129 (55.7%)	290 (72.9%)	583 (55.8%)	256 (36.7%)		
Current disease progression status	Acute phase	1 (0.0%)	0 (0.0%)	0 (0.0%)	1 (0.1%)	139.002	<0.001*
	Remission phase	55 (2.6%)	3 (0.8%)	21 (2.0%)	31 (4.4%)		
	Rehabilitation phase	842 (39.3%)	83 (20.9%)	394 (37.7%)	365 (52.3%)		

χ^2^ = value of χ^2^ statistics for chi-square test. *p < 0.05 is statistically significant.

### 3.2 Latent profile analysis of occupational fatigue

**Figure 1:**
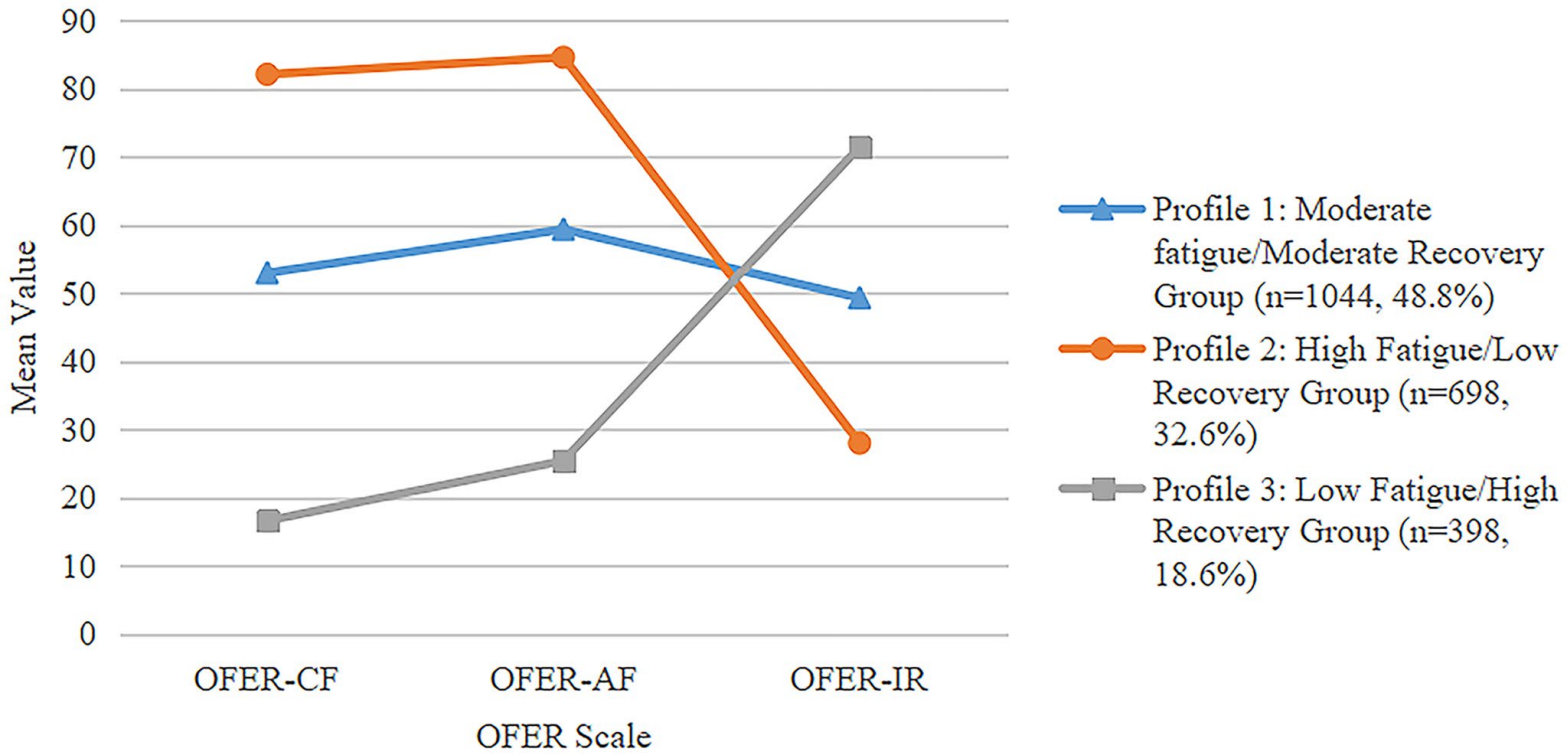
Latent profile plot of estimated means for the occupational fatigue.

Commencing with the initial model, one to five profile classes were progressively modeled, and the fit indices for each latent profile structure are shown in Table 2. Results indicated that AIC, BIC and aBIC decreased with an increasing number of latent profiles. Considering model fit tests, parsimony, and interpretability, the 3-profile solution demonstrated the overall best fit, as indicated by the highest entropy values and a significant LMRT result (*P* <0.05). The three identified profiles and the predicted mean values for each dimension of the OFER scale from the LPA model are depicted in Figure. 1. Based on the patterns of probability responses and comparison of average values of grouping variables, the identified profiles were named as follows: profile 1 with moderate mean values of acute fatigue and chronic fatigue, along with inter-shift recovery, labeled as the ‘moderate fatigue/moderate recovery group’ (n = 1,044, 48.8%); profile 2 representing the highest means of acute fatigue and chronic fatigue, coupled with the lowest means of inter-shift recovery, named the ‘high fatigue/low recovery group’ (*n* = 698, 32.6%); profile 3 displaying the lowest values of acute fatigue and chronic fatigue, accompanied by the highest levels of inter-shift recovery, was called as the ‘low fatigue/high recovery group’ (*n* = 398, 18.6%). These profiles provide a nuanced understanding of the diverse experiences of occupational fatigue among Chinese nurses in the early stage of the post-COVID-19 era.

**Table 2:** Fit indices for the latent profile analysis.

No. of profiles	K	AIC	BIC	aBIC	Entropy	LMRT *P-*value	BLRT *P-*value	Category probability
1	6	59000.844	59034.856	59015.793	–	–	–	1.00
2	10	56704.380	56761.065	56729.294	0.803	0.0000	0.0000	0.33/0.67
**3**	**14**	**55582.455**	**55661.815**	**55617.335**	**0.825**	**0.0000**	**0.0000**	**0.49/0.33/ 0.18**
4	18	55274.400	55376.434	55319.246	0.775	0.1226	0.0000	0.17/0.14/0.31/0.38
5	22	55123.622	55248.330	55178.433	0.766	0.0028	0.0000	0.14/ 0.08/0.36/0.29/0.13

Bold face indicates the best-fitting model.

### 3.3 Univariate analysis of latent profiles of nurses’ occupational fatigue

Univariate analysis was performed to compare the identified profiles of occupational fatigue against socio-demographic and work-related characteristics, organized by group (Table 1). Statistical differences were observed across different groups on marital status, education, hospital sites, hospital grades, occupation, practice unit, average hours of weekly work, number of night shifts per month, direct provision of care to patients with COVID-19, suffering from COVID-19 related symptoms, and current disease progression status. Table 3 further illustrates that all dimensions of the ERI model, encompassing extrinsic effort, reward, overcommitment and the extrinsic effort/reward ratio, exhibited significant differences among the latent fatigue profiles. This comprehensive univariate analysis provides insights into how various socio-demographic and work-related factors, as well as components of the ERI model, contribute to the distinctive profiles of occupational fatigue among Chinese nurses in the early stage of the post-COVID-19 era.

**Table 3:** Differences in the ERI model among latent fatigue profiles.

Variables	Groups	Means ± SD/N (%)	Low fatigue/ High recovery (*n* = 398)	Moderate fatigue/ Moderate recovery (*n* = 1,044)	High fatigue/ Low recovery (*n* = 698)	*F*/*χ^2^*	*P-*value
Extrinsic effort		18.04 ± 6.27	10.86 ± 4.19	17.16 ± 4.53	23.45 ± 4.48	1052.454	<0.001*
Reward		38.71 ± 5.15	39.17 ± 5.06	38.90 ± 4.65	38.16 ± 5.84	5.439	0.004*
Overcommitment		16.82 ± 3.49	13.29 ± 3.06	16.46 ± 2.56	19.37 ± 2.88	547.937	<0.001*
Extrinsic effort/	>1	661 (30.9%)	7 (1.8%)	203 (19.4%)	451 (64.6%)	594.138	<0.001*
Reward ratio	≤1	1,479 (69.1%)	391 (98.2%)	841 (80.6%)	247 (35.4%)		

ERI, Effort-Reward Imbalance; SD, Standard Deviation. F = value of F statistics for a one-way analysis of variance (ANOVA) test, χ^2^ = value of χ^2^ statistics for chi-square test. *P < 0.05 is statistically significant.

### 3.4 Multinomial logistic regression analysis for latent profiles of occupational fatigue in nurses

All significant factors identified in the univariate analysis were taken into the multinomial logistic regression to evaluate their relationship with profile membership (Table 4). The effect size is represented by the Odds Ratio (OR) and its corresponding 95% confidence interval. An odds ratio exceeding 1.0 indicates that nurses within a specific category have an increased risk of being associated with a particular fatigue profile than their counterparts, while less than 1.0 suggests a decreased risk. In comparison with the low fatigue/high recovery group: participants working more than 5 night shifts per month (OR: 1.867, 95%CI: 1.188–2.935 and OR: 2.847, 95%CI: 1.592–5.091), suffering from COVID-19 related symptoms (OR: 2.211, 95%CI: 1.545–3.162 and OR: 3.828, 95%CI: 2.490–5.884), demonstrating higher extrinsic effort (OR: 1.318, 95%CI: 1.261–1.378 and OR: 1.634, 95%CI: 1.536–1.739) and overcommitment scores (OR: 1.303, 95%CI: 1.223–1.387 and OR: 1.614, 95%CI: 1.489–1.749) had higher odds of belonging to the moderate fatigue/moderate recovery group and the high fatigue/low recovery group, respectively. On the contrary, nurses who got married (OR: 0.473, 95%CI: 0.320–0.700) and held a master’s degree or above (OR: 0.149, 95%CI: 0.030–0.735) had lower odds of being in the moderate fatigue/moderate recovery group than their counterparts. Similarly, married nurses (OR: 0.512, 95%CI: 0.318–0.824) were also less likely to belong to the high fatigue/low recovery group compared to the low fatigue/high recovery group. These findings shed light on the factors influencing the membership of nurses in different occupational fatigue profiles, providing valuable insights for targeted interventions and support strategies.

**Table 4:** Multinomial logistic regression results predicting profile membership.

Variables	Moderate fatigue/Moderate recovery group			High fatigue/Low recovery group		
	B	OR (95%CI)	*P*-value	B	OR (95%CI)	*P*-value
Marital status						
Single	Ref			Ref		
Married	−0.749	0.473 (0.320–0.700)	0.000*	−0.670	0.512 (0.318–0.824)	0.006*
Divorced/widowed	−1.444	0.236 (0.089–0.624)	0.054	−0.686	0.503 (0.146–1.741)	0.278
Education						
Diploma	Ref			Ref		
Bachelor’s degree	0.054	1.056 (0.746–1.494)	0.759	0.181	1.198 (0.774–1.856)	0.418
Master’s degree or above	−1.904	0.149 (0.030–0.735)	0.019*	−1.208	0.299 (0.049–1.831)	0.192
Number of night shifts per month						
0	Ref			Ref		
1 ~ 4	0.384	1.468 (0.915–2.356)	0.111	0.523	1.688 (0.910–3.131)	0.097
≥5	0.625	1.867 (1.188–2.935)	0.007*	1.406	2.847 (1.592–5.091)	0.000*
Currently suffering from COVID-19 related symptoms						
Yes	0.793	2.211 (1.545–3.162)	0.000*	1.342	3.828 (2.490–5.884)	0.000*
No	Ref			Ref		
Extrinsic effort	0.276	1.318 (1.261–1.378)	0.000*	0.491	1.634 (1.536–1.739)	0.000*
Reward	−0.027	0.973 (0.933–1.015)	0.206	−0.007	0.993 (0.944–1.044)	0.780
Overcommitment	0.264	1.303 (1.223–1.387)	0.000*	0.479	1.614 (1.489–1.749)	0.000*

Reference group: Low fatigue/High recovery group. ^*^P<0.05 is statistically significant.

## 4 Discussion

### 4.1 The status of occupational fatigue among Chinese nurses in the early stage of post-COVID-19 era

The findings of this study contribute to the existing literature that identified three distinct profiles of occupational fatigue in Chinese nurses, including the low fatigue/high recovery group, moderate fatigue/moderate recovery group, and high fatigue/low recovery group. Examining the proportions of respondents in each group, it is evident that the vast majority of nurses reported moderate to high levels of occupational fatigue during the early stage of the post coronavirus era (Figure 1). In comparison to prevalence rates observed in other countries, such as 53.4% in the United States (33), 55% in Australia (22), and 62% in Korea (34), the prevalence of occupational fatigue among nurses in this study, at 81.4%, appears to be relatively high. This could be attributed to the challenging medical environment in China. According to the World Health Organization (WHO) State of the World’s Nursing Report 2020 (SOWN), the nursing personnel density in China remains significantly lower compared to developed countries. The density of nurses per 10,000 population is approximately 26.6 in China, while it is 145.5 in the United States, 121.7 in Japan, and 116.8 in Australia (35). Additionally, the pandemic has led to a notable increase in the number of nurses leaving the profession globally, including Chinese nurses (36). This, combined with the high infection rates among healthcare workers, could contribute to the magnification and exacerbation of nursing shortages in the post-COIVD-19 era. Nurses who remain in their roles may face heavier workloads, longer working hours and greater occupational stress to meet the escalating care demands for patients. Moreover, the influence of traditional Chinese culture, which emphasizes collectivism and self-sacrifice, is deeply ingrained in the social orientation of Chinese people (37). In collectivist cultures, there is a strong emphasis on the interdependent relationship between individuals and their groups, with a prevailing belief that the interests of the collective take precedence over those of the individual. Within this cultural framework, nurses may feel a complete responsibility for their patients and unit, and engage in excessive work for patients’ well-being, potentially leading to physical and mental exhaustion. Furthermore, the emphasis on harmony within collectivist cultures may result in nurses’ reluctance to convey their genuine emotions when confronted with pressure and conflict. Over time, this suppression can subsequently lead to the development of fatigue. The cultural value of self-sacrifice has also been increasingly reinforced in this context. Particularly when confronted with difficulties and challenges, such as the sudden surge in patient numbers and a marked increase in infections among healthcare workers, nurses may tend to overlook their personal needs in favor of pursuing collective success. This inclination often leads them to sacrifice their time and energy to fulfill their assigned tasks even when fatigued (38). Understanding these cultural nuances is crucial for developing targeted interventions and support mechanisms to address occupational fatigue in the Chinese nursing workforce.

### 4.2 The influencing factors of occupational fatigue profile membership in Chinese nurses following the post-COVID-19 era

The regression analysis revealed that, compared with the low fatigue/high recovery group, nurses working more than 5-night shifts per month were more likely to belong to the moderate fatigue/moderate recovery group and high fatigue/low recovery group, respectively (Table 4). Human beings exhibit diurnality, characterized by the release of higher levels of adrenaline and corticosteroids during the day and increased secretion of sleep-dependent hormones at night (39). Shift work that disrupts these circadian rhythms can impact sleep quality and contribute to higher levels of occupational fatigue. It is understandable that nurses working more night shifts are associated with an increased likelihood of experiencing worse sleep quality and higher levels of occupational fatigue compared to day shift workers. This aligns with findings from other studies suggesting that night nurses may experience reduced efficiency and increased fatigue due to decreased levels of adrenaline and corticosteroids during night shifts (10, 17). The demanding nature of hospital nursing emphasizes the importance of prioritizing the maintenance of optimal physical and mental health for individuals exposed to disasters. This is crucial not only during but also before and after such events, enabling effective responses (40). While compelling evidence suggests that COVID-19 may have medium-and long-term consequences, impacting the quality of life in affected individuals (41, 42). Nurses who have not fully recovered from COVID-19, yet return to work due to the lack of human resources, understandably face higher levels of occupational fatigue. Thus, it is crucial for nursing managers to undertake a thorough evaluation of both the physical and mental well-being of nurses prior to assigning them to night shifts. Simultaneously, they must enhance efforts to optimize shift scheduling by ensuring sufficient staffing levels for nighttime duties, reducing the length of night shifts, providing appropriate rest periods and a comfortable resting environment for nurses during their shifts, and regularly gathering feedback concerning shift arrangements to support continuous improvement.

The logistic regression analysis also highlighted that higher scores in extrinsic effort and overcommitment were significant risk factors for moderate fatigue/moderate recovery group and high fatigue/low recovery group, respectively, with the exception of the reward dimension in the ERI model (Table 4). This observation is likely connected to the prevailing practice environment for Chinese nurses. These professionals bear substantial workloads and exert considerable extrinsic effort in the performance of their duties. However, the rewards they receive may be perceived as somewhat disproportionate. This could include relatively low pay, inadequate respect from patients and physicians, and limited opportunities for career advancement and professional development within their workplace (43). The imbalanced working conditions, characterized by high effort but low reward, contribute to occupational stress, further amplifying levels of fatigue among nurses. Furthermore, the influence of Chinese traditional culture, as mentioned earlier, instills a personality trait of overcommitment in nurses. Consistent with other research findings, higher levels of overcommitment in nurses are associated with a greater likelihood of experiencing severe occupational fatigue (43, 44). Therefore, it is imperative for policymakers and hospital administrators to implement reward mechanisms for nurses. This includes considerations for higher compensation, increased opportunities for career progression and continuing education, and the creation of a safe and supportive working environment. Such measures can bolster nurses’ professional identity and job satisfaction, striving to achieve a balance between extrinsic effort and overcommitment. This, in turn, contributes to mitigating occupational fatigue among Chinese nurses.

The study results also indicated that married nurses had lower odds in the moderate fatigue/moderate recovery group and high fatigue/low recovery group compared to their counterparts (Table 4). One possible explanation for this finding is that married nurses benefit from more supportive relationships within their families, which could aid in the recovery from work-related fatigue (22). However, it’s worth noting that this is a controversial issue. Some other studies have suggested that married individuals exhibit significantly higher levels of occupational fatigue than those with other marital status (43, 45). It can be argued that, as a result of prevailing social and cultural conventions, married nurses in China are not only required to diligently fulfill their duties in the hospital to deliver superior service, but also bear the responsibility of managing household chores, attending to the needs of their children, and providing support to older adult family members (44). The challenges associated with reconciling work–family conflicts may exacerbate fatigue. The present results should be interpreted cautiously, and future research is warranted to explore this aspect further. In any case, it is imperative to establish a more supportive work environment for married nurses. For instance, family members could assume a greater share of domestic responsibilities, such as household chores and childcare, to alleviate the burden of family caregiving and mitigate potential family-work conflicts. Additionally, supervisors should exhibit increased empathy and understanding, while colleagues ought to engage in more collaborative and cooperative practices.

The study also found that nurses holding a master’s degree or above were less likely to belong to the high fatigue/low recovery group than their peers with diploma and bachelor’s degrees (Table 4). This could be elucidated by the overall low educational level in Chinese nurses, where over 90% possess diploma and bachelor’s degrees, and only a small minority (1%) holds a master’s degree or above (35). Consequently, this group of highly educated nurses receives more attention and is often engaged in nursing teaching, research, and management work. They are less likely to be involved in clinical roles that require night shifts (46). As a result, they tend to experience less fatigue compared to nurses with lower educational levels. This highlights the importance of education and career development opportunities in mitigating occupational fatigue among individuals with lower educational attainment.

### 4.3 Limitations and future research

Several limitations should be acknowledged when interpreting the study findings.

#### 4.3.1 Sampling methodology

The use of convenience sampling and online recruitment may introduce selection bias, limiting the generalizability of the results. Future research could benefit from employing random sampling techniques to enhance representativeness.

#### 4.3.2 Subjective measures

While the instruments used in the study demonstrated robust psychometric properties, reliance on self-reported and subjective variables may introduce recall-bias and self-reporting limitations. Future studies might consider incorporating objective indicators, such as wearable devices, to provide a more accurate and unbiased assessment of occupational fatigue.

#### 4.3.3 Cross-sectional design

The study’s cross-sectional design restricts the ability to establish causal relationships among the study variables. Future research should incorporate prospective study designs to better elucidate temporal associations and causal pathways.

#### 4.3.4 Long-term effects of COVID-19

Given the evolving nature of the COVID-19 pandemic, the study focused on the early stage of the post-COVID-19 era. Long-term effects on occupational fatigue and other related factors may emerge over time. Therefore, it is necessary to carry out longitudinal studies to explore the sustained impact of the pandemic on healthcare professionals and to assess the long-term efficacy of the proposed interventions on fatigue mitigation.

## 5 Conclusion

The current study confirmed three distinct profiles of occupational fatigue among Chinese nurses through the latent profile analysis, which included low fatigue/high recovery group, moderate fatigue/moderate recovery group and high fatigue/low recovery group. The majority of nurses revealed moderate to high levels of occupational fatigue in the early stage of the post-COVID-19 era. Several factors were found to be significantly associated with the profile membership, including marital status, educational attainment, night shift frequency, COVID-19 symptoms, extrinsic effort, and overcommitment. These findings provide valuable insights for health policymakers and nursing administrators to optimize human resource allocation in the aftermath of the post-COVID-19 era. Furthermore, the results underscore the importance of developing targeted fatigue alleviation interventions for specific groups of nurses, aiming to effectively to address the challenges posed by occupational fatigue in the face of future public health disasters.

## Data Availability

The original contributions presented in the study are included in the article/supplementary material, further inquiries can be directed to the corresponding authors.
